# Pbs2 regulates late‐stage macroautophagy in *Saccharomyces cerevisiae*

**DOI:** 10.1002/ame2.70042

**Published:** 2025-06-16

**Authors:** Jianing Song, Haolin Zhang, Xingyu Cao, Zizhang Ren, Chao Tian, Miao Jia, Meiling Wu, Xiaoli Wang, Juan Wang

**Affiliations:** ^1^ College of Chemistry and Life Science Beijing University of Technology Beijing China

**Keywords:** autophagy, Hog1, MAPK, Pbs2

## Abstract

Autophagy is crucial for maintaining cellular homeostasis and is linked to various diseases. In *Saccharomyces cerevisiae*, the Polymyxin B Sensitivity 2 (Pbs2) protein is a member of the mitogen‐activated protein kinase (MAPK) family and plays a role in mitophagy. To explore the potential role of Pbs2 in macroautophagy, we engineered wild‐type and *PBS2*‐deficient cells using plasmid construction and yeast transformation techniques, followed by a series of autophagy assays. First, after nitrogen starvation, the levels of autophagic activity were evaluated with the classical GFP‐Atg8 cleavage assay and the Pho8Δ60 activity assay at different time points. Deleting *PBS2* significantly decreased both GFP‐Atg8 protein cleavage and Pho8Δ60 activity, indicating that Pbs2 is essential for macroautophagy. Furthermore, the influence of Pbs2 on macroautophagy was shown to be independent of Hog1, a well‐known downstream factor of Pbs2. Second, the Atg8 lipidation assay demonstrated that Atg8 lipidation levels increased upon *PBS2* deletion, suggesting that Pbs2 acts after Atg8 lipidation. Third, the proteinase K protection assay indicated that the loss of *PBS2* led to a higher proportion of closed autophagosomes, implying that Pbs2 impacts the later stages of macroautophagy following autophagosome closure. In conclusion, Pbs2 regulates the late stages of macroautophagy induced by nitrogen starvation.

## Graphical abstract

The MAPK family member Pbs2 and its role in autophagy. Macroautophagy consists of five key steps: the initiation at the pre‐autophagosomal structure (PAS), elongation of the isolation membrane (IM), maturation of the autophagosome, fusion of autophagosomes with vacuoles, and degradation of autophagic bodies. Pbs2 is involved in macroautophagy, affecting the stages that follow the closure of the autophagosome.

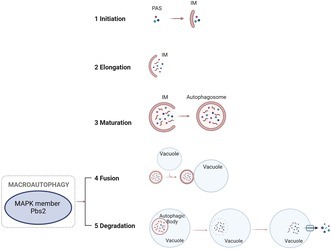

## 1 INTRODUCTION

Autophagy is a process in which substrate proteins and organelles are enveloped by a double‐membrane structure and transported to the vacuole or lysosome for degradation. This process is crucial for maintaining cellular homeostasis and responding to environmental challenges.^1^, ^2^ In *Saccharomyces cerevisiae*, under nutrient‐rich conditions, autophagy remains at normal levels. But autophagy is activated when nutrients like glucose, nitrogen, and carbon become scarce.^3^ During autophagy induction, autophagy‐related proteins (Atgs) are recruited to the pre‐autophagosomal structure (PAS) to form a double‐membrane structure called the phagophore or isolation membrane (IM).^4^, ^5^ As the IM expands, more Atg proteins gather at this site. Once the IM extends sufficiently, it seals to form an autophagosome.^6^ The autophagosome then fuses with the vacuole to degrade target proteins.

Mitogen‐activated protein kinases (MAPKs) serve as primary intracellular signal transduction mediators in eukaryotic cells.^7^ These kinases activate in response to external environmental stresses, orchestrating cellular responses, including proliferation, differentiation, apoptosis, and autophagy.^8^, ^9^ In *S. cerevisiae*, five distinct MAPK pathways exist, each performing specific functions.^7^ Importantly, the HOG pathway members Pbs2 MAP2K and its substrate Hog1 MAPK^10^, ^11^ are essential for mitophagy.^12^, ^13^ Mitophagy is a specialized form of autophagy where autophagosomes selectively encapsulate and degrade mitochondria.^14^, ^15^ When mitophagy is triggered, the mitochondria‐specific resident protein Atg32 associates with Atg11, which acts as an adaptor protein for selective autophagy.^13^ This interaction leads to the phosphorylation of Atg32, which promotes the process of mitophagy. Studies have indicated that Pbs2 and Hog1 indirectly modulate the phosphorylation of Atg32, thereby contributing to mitophagy.^12^, ^13^, ^16^ This finding prompted us to investigate whether Pbs2 or Hog1 is also involved in macroautophagy. This study demonstrates that Pbs2 is involved in macroautophagy induced by nitrogen starvation. However, this effect is not mediated by Hog1. Additionally, the stage at which PBS2 participates in macroautophagy is after the autophagosome closure.

## 2 MATERIALS AND METHODS

### 2.1 Strains, plasmids and primers

The yeast strains utilized in this study are listed in Table 1. The *pho8Δ60* plasmid was obtained from Danial J. Klionsky's lab, while the Prs416‐GFP‐ATG8 plasmid was sourced from Susan Ferro‐Novick's lab. The primers used in this study are detailed in Table 2, and they were synthesized and sequenced by Beijing Qingke Biotechnology.

**TABLE 1:** *Saccharomyces cerevisiae* strains used in this study.

Strain	Genotype	Source
BY4742	*MATα his3Δ1; leu2Δ0; lys2Δ0; ura3Δ0*	Invitrogen
*pbs2Δ*	BY4742; *pbs2Δ::kanMX6*	Invitrogen
WT [GFP‐Atg8]	BY4742; *ura3::GFP‐ATG8‐URA3*	This study
*pbs2Δ* [GFP‐Atg8]	BY4742; *pbs2Δ:: kanMX6; ura3::GFP‐ATG8‐URA3*	This study
WT [*pho8Δ60*]	BY4742; *pho8::pho8Δ60‐URA3*	This study
*pbs2Δ*[*pho8Δ60*]	BY4742; *pbs2Δ:: kanMX6; pho8Δ60Δ::URA3*	This study
*hog1Δ*	BY4742; *hog1Δ::kanMX6*	Invitrogen
*hog1Δ* [GFP‐Atg8]	BY4742; *hog1Δ:: kanMX6; ura3::GFP‐ATG8‐URA3*	This study
*hog1Δ*[*pho8Δ60*]	BY4742; *hog1Δ:: kanMX6; pho8Δ60Δ::URA3*	This study
WT [Ape1‐RFP;GFP‐Atg8]	BY4742; *ura3::GFP‐ATG8‐URA3; leu2::Ape1‐RFP‐LEU2*	This study
*pbs2Δ*[Ape1‐RFP;GFP‐Atg8]	BY4742; *pbs2Δ:: kanMX6; ura3::GFP‐ATG8‐URA3; leu2::Ape1‐RFP‐LEU2*	This study
WT [Ape1‐RFP;Atg1‐GFP]	BY4742; *ura3::ATG1‐GFP‐URA3; leu2::Ape1‐RFP‐LEU2*	This study
*pbs2Δ*[Ape1‐RFP;Atg1‐GFP]	BY4742; *pbs2Δ:: kanMX6; ura3::ATG1‐GFP‐URA3; leu2::Ape1‐RFP‐LEU2*	This study
WT [Ape1‐RFP;Atg17‐GFP]	BY4742; *ura3::ATG17‐GFP‐URA3; leu2::Ape1‐RFP‐LEU2*	This study
*pbs2Δ*[Ape1‐RFP;Atg17‐GFP]	BY4742; *pbs2Δ:: kanMX6; ura3::ATG17‐GFP‐URA3; leu2::Ape1‐RFP‐LEU2*	This study
WT [Ape1‐RFP;Atg18‐GFP]	BY4742; *ura3::ATG18‐GFP‐URA3; leu2::Ape1‐RFP‐LEU2*	This study
*pbs2Δ*[Ape1‐RFP;Atg18‐GFP]	BY4742; *pbs2Δ:: kanMX6; ura3::ATG18‐GFP‐URA3; leu2::Ape1‐RFP‐LEU2*	This study

**TABLE 2:** Primers used in this study.

Primer	Sequence
PBS2‐forward	TGTGGGTACACGTTTCACAGAAC
PBS2‐reverse	GTATTCGCCGCATCATTTCC
KANMX6‐forward	TGATTTTGATGACGAGCGTAAT
KANMX6‐reverse	CTGCAGCGAGGAGCCGTAAT
pho8Δ60‐forward	CGCAATTAACCCTCACTAAAGGGAACAAA
pho8Δ60‐reverse	GCGTAATACGACTCACTATAGGGCGAATTG
pho8Δ60‐D‐forward	GGAGGAGAACAATAACCGCACC
pho8Δ60‐D‐reverse	TCGGTCCCGTAGCTCACTGAC

### 2.2 Western blotting

The prepared protein samples were mixed with 2 × sodium dodecyl sulfate (SDS) loading buffer, denatured by boiling at 100°C for 10 min, and detected by Western blot. The samples were loaded onto 10% or 15% SDS polyacrylamide gels (SDS‐PAGE) and were electrophoretically separated using a Tris running buffer (25 mM Tris, 250 mM glycine, 0.1% w/v SDS). Gels were transferred to polyvinylidene fluoride (PVDF) membranes using Tris‐based transfer buffer (25 mM Tris base, 0.2 M glycine, 20% methanol, pH 8.3). The membranes were incubated for 1 h in blocking buffer (5% nonfat milk in TBST) and subsequently in primary antibodies overnight at 4°C. The membranes were rinsed in TBST three times and incubated with secondary antibodies at room temperature for 1 h. Next, membranes were rinsed three times using TBST and detected using ECL (MerckMillipore WBKLS0500) and a chemiluminescence image analysis system (Tanon 5200). Antibodies used in this study were anti‐GFP Polyclonal antibody (1: 3000, 50430‐2, proteintech), anti‐ATG8 Polyclonal antibody (1: 2000, AB4753, Abcam), and HRP‐conjugated goat anti‐rabbit IgG (H + L) (1: 5000, SA00001, proteintech).

### 2.3 Culture conditions and nitrogen starvation‐induced macroautophagy

The yeast basic medium used was YPD, which consists of 1% yeast extract, 2% peptone, and 2% glucose. Additionally, selective nutrient‐deficient media known as SC‐were used; they contain 0.67% yeast nitrogen base, 2% glucose, and a mixture of amino acids. The names of the deficient nutrients are indicated after ‘SC’. Furthermore, an SD‐N medium was utilized, consisting of 0.17% yeast nitrogen base without amino acids and ammonium sulfate, along with 2% glucose. To produce a solid medium, agar was added to a final concentration of 2%. The strain was grown to logarithmic growth period in either a basal medium or a selective nutrient‐deficient medium. It was then centrifuged at 3000 *g* for 2 min, washed with PBS, and resuspended in SD‐N. The cells were starved at various time points based on the experimental requirements to induce macroautophagy.

### 2.4 GFP‐Atg8 cleavage assay

Cells expressing GFP‐Atg8 were grown overnight to OD_600_ = 0.8–1.0 at 30°C in SC‐Ura medium. Then cells were centrifuged at 3000 *g* for 2 min. The precipitates were washed twice using 0.1 M PBS and resuspended with SD‐N mdium. Next cells were cultured at 30°C. Cells at 2 OD_600_ units were collected after 0, 1, 2 and 4 h. Cells were washed with PBS for twice and the precipitates were resuspended with 200 μL ddH_2_O and 200 μL 0.2M NaOH. The samples were incubated on ice for 10 min and then centrifuged at 12 000 rpm 4°C for 5 min. The supernatant was removed and the pellet resuspended in 80 μL SDS loading buffer. The samples were denatured and detected by Western blot.

### 2.5 Pho8Δ60 activity assay

Pho8Δ60 experiments were performed as previously described.^17^ Briefly, strains were cultured in SC‐Ura medium to OD_600_ = 0.8 and swapped for SD‐N culture after two rinses in PBS. Cells at 2 OD_600_ units were collected at each starvation time point, resuspended with appropriate amounts of glass beads and ice‐cold lysis buffer (20 mM PIPES, pH 7.2, 0.5% Triton X‐100.50 mM KCL, 100 mM potassium acetate, 10 mM MgSO_4_,10 μm ZnSO_4_, and 1 mM PMSF). The protein was extracted after vortexing and centrifugation, and then 400 μL reaction solution (1.25 mM p‐nitrophenyl phosphate, 250 mM Tris–HCl, pH 8.5, 0.4% Triton X‐100, 10 mM MgSO_4_, and 10 μm ZnSO_4_) was added to the supernatant, and the mixed solution was incubated at 37°C for 20 min. The reaction was quenched by adding 500 μL stop buffer (1 M glycine/KOH, pH 11.0) and the OD_400_ value was determined by a spectrophotometer (Bio‐Tek Epoch).

### 2.6 GFP‐Atg8 proteinase protection assay

Proteinase protection assay was performed as previously described.^18^ GFP‐ATG8‐expressing cells were grown to the log phase in SC‐Ura medium and were transferred to SD‐N for starvation for 3 h after PBS rinsing. Cells at 50 OD_600_ units were collected to prepare spheroplasts. The spheroplasts were washed with SP buffer (1 M sorbitol, 20 mM PIPES, pH 6.8) and then lysed with 1.8 mL PS200 buffer (20 mM PIPES, pH 6.8, 200 mM sorbitol, 5 mM MgCl_2_) on ice for 10 min. Unbroken cells were removed by a centrifugation at 300× *g* and the supernatant was subjected to a 5000× *g* spin. The resulting pellet, P5, was re‐suspended in buffer and used for proteinase K and Triton X‐100 treatments. Following the protease treatment, proteins were precipitated with TCA for immunoblot analysis using an anti‐GFP antibody to determine the levels of degraded and non‐degraded proteins.

### 2.7 Fluorescence microscopy

Cells were grown to log phase in YPD medium. Then cells were resuspended with SD‐N after PBS washing. After 1 h culture for macroautophagy induction, cells were dripped onto the slide. The samples were observed with a confocal microscope (Nikon AX R) under the 60 × oil‐immersion objective. Images were exported with the NIS Elements Viewer software and processed in the Image J software.^4^ Ratios from all the cells (*n* > 100, three biological repeats) were analyzed.

### 2.8 Atg8 lipidation assay

Cells were grown overnight to OD_600_ = 0.8–1.0 at 30°C in YPD medium. Then cells were centrifuged at 3000 *g* for 2 min. The precipitates were washed twice using 0.1 M PBS and resuspended with SD‐N mdium. After that, cells were cultured at 30°C and collected at 2 OD_600_ units at 0, 1, 2 and 4 h. Cells were washed twice with PBS and the precipitates were resuspended with 200 μL ddH_2_O and 200 μL 0.2 M NaOH. The samples were incubated on ice for 10 min, and centrifuged at 12 000 rpm at 4°C for 5 min. The supernatant was removed and the pellet was resuspended with 80 μL SDS‐PAGE protein loading buffer. To separate Atg8‐PE from Atg8, lysates of cells were examined on a 15% SDS‐PAGE gel with 6 M urea, and blots were probed with an anti‐Atg8 antibody.

## 3 RESULTS

### 3.1 Pbs2 regulates nitrogen starvation‐induced macroautophagy

**FIGURE 1:**
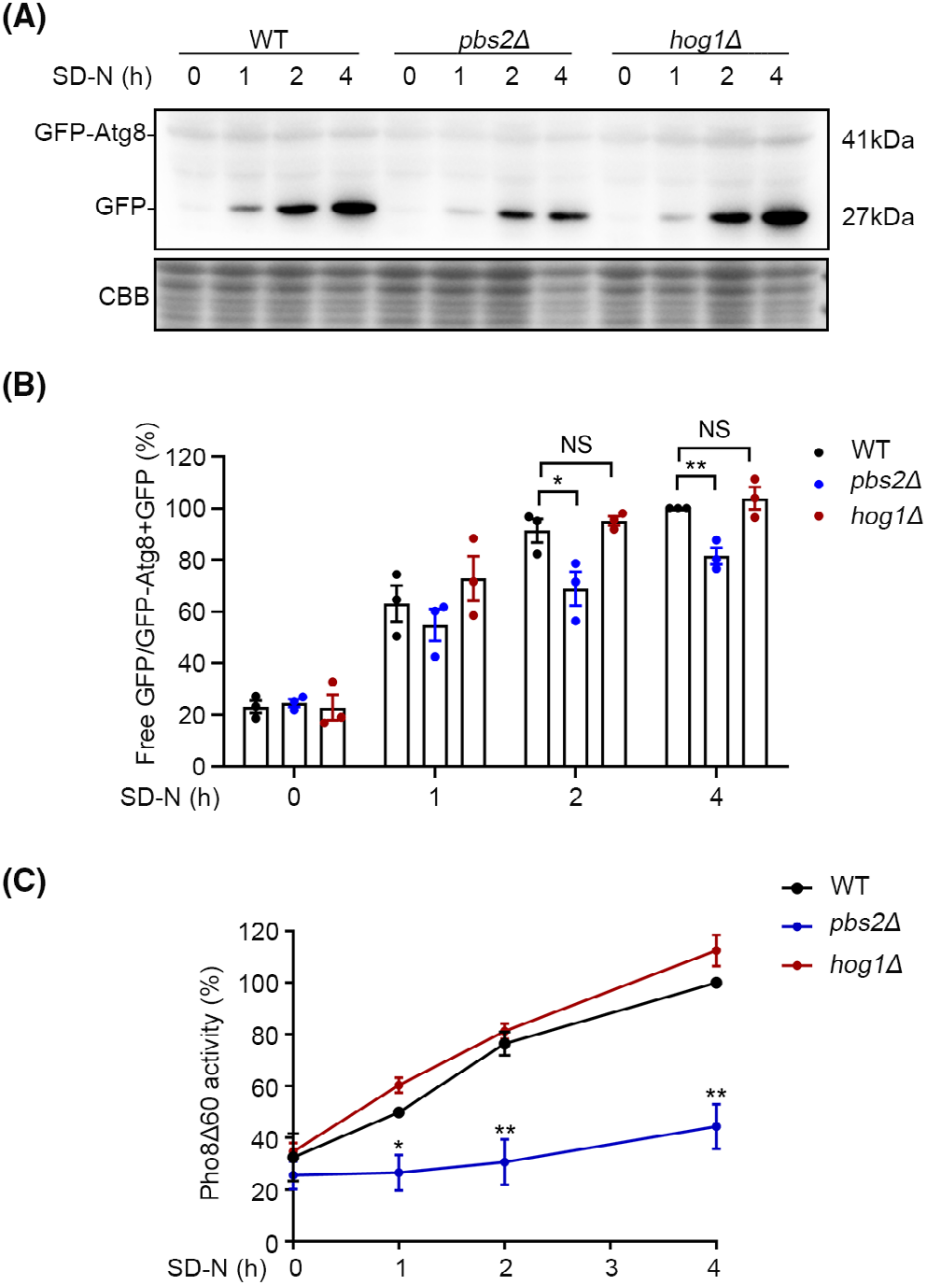
Pbs2 plays a role in macroautophagy triggered by nitrogen starvation. (A) GFP‐Atg8 cleavage processing in WT, *pbs2Δ*, and *hog1Δ* were tested. The strains were cultured in YPD to OD_600_ of 1 and then transferred to SD‐N for starvation for 1, 2, or 4 h at 30°C. Samples were detected by Coomassie staining (CBB) and Western blot with an anti‐GFP antibody. (B) The ratio of free GFP to total GFP was calculated. Error bars represent SEM; *n* = 3; NS, not significant; *p < 0.05; **p < 0.01, unpaired Student's *t* test. (C) Pho8Δ60 activity was measured in WT, *pbs2Δ*, and *hog1Δ* cells. The strains were starved at 30°C for 0, 1, 2 or 4 h. The Pho8Δ60 activity for WT at 4 h was set as 100%. Error bars represent SEM; *n* = 3; *p < 0.05; ***p < 0.001, unpaired Student's *t* test.

Pbs2 and Hog1 are members of the MAPK kinase family and are involved in selective autophagy, particularly in mitophagy.^12^, ^13^ However, whether Pbs2 or Hog1 also participates in nitrogen starvation‐induced macroautophagy is still unknown. To address this question, first, we established and verified the *pbs2Δ* and *hog1Δ* strains (Figure S1A,B). Next, we explored the role of Pbs2 or Hog1 in macroautophagy using the GFP‐Atg8 cleavage assay^19^ and the Pho8Δ60 activity assay,^17^ which measure autophagic activity. During macroautophagy induction, GFP‐Atg8 fusion protein is delivered to the yeast vacuole, where Atg8 is degraded while GFP remains relatively intact due to its resistance to proteolysis. Thus, the extent of GFP‐Atg8 cleavage serves as a reliable indicator of autophagic activity.^20^ Autophagic activity was assessed by Western blot analysis using anti‐GFP antibodies, measuring the ratio of free GFP to total GFP. In wild‐type (WT) cells under nutrient‐rich conditions, GFP‐Atg8 was predominantly present in the form of GFP‐Atg8. As the duration of nitrogen starvation increased, the proportion of free GFP rose sharply (Figure 1A). However, in the *pbs2Δ* but not in the *hog1Δ* cells, GFP‐Atg8 did not significantly cleave into GFP after 1, 2, or 4 h of treatment. Quantitative analysis from three independent experiments showed a significant reduction in GFP‐Atg8 cleavage in *pbs2Δ* cells compared to wild‐type cells (Figure 1B).

To further confirm whether there is an macroautophagy defect in the mutant strain, we employed the pho8Δ60 activity assay.^19^ Under normal conditions in yeast, pho8 is transported from the endoplasmic reticulum (ER) to the vacuole, where it is cleaved into the active form by resident proteases. The Pho8Δ60 protein, which lacks the first 60 N‐terminal amino acids, is transported to the vacuole via the macroautophagy pathway only. Thus, measuring protease activity for Pho8Δ60 serves as a marker of autophagic activity.^17^, ^21^ The Pho8Δ60 gene fragment was recombined into the yeast genome via yeast transformation, and genotypic validation was performed (Figure S1C,D). In WT cells, enzyme activity increased with prolonged starvation (Figure 1C). Consistent with the results of the GFP‐Atg8 cleavage assay, under different durations of starvation, both *hog1Δ* and WT cells exhibited a comparable increase in autophagic activity. However, the autophagic activity in *pbs2Δ* cells did not show a significant increase (Figure 1C). Together, these results show that Pbs2 is required to nitrogen starvation‐induced macroautophagy in *S. cerevisiae* and the effect of Pbs2 on macroautophagy is independent of Hog1.

### 3.2 Pbs2 functions after the stage of autophagosome closure

**FIGURE 2:**
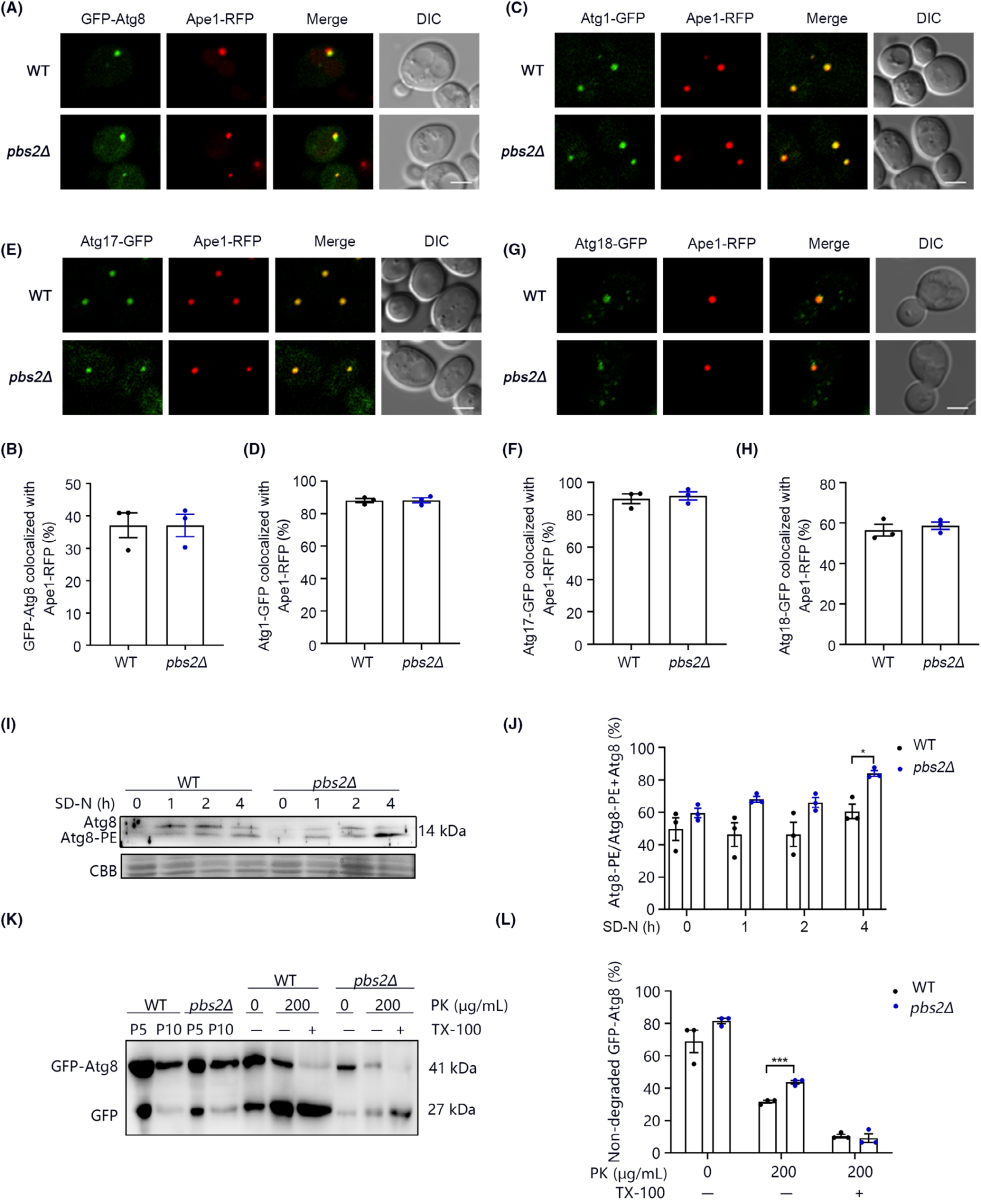
Validation of Pbs2‐associated autophagic stages. (A, B) The translocation of GFP‐Atg8 and Ape1‐RFP were examined in WT and *pbs2Δ* cells 1 h after nitrogen starvation. (A) Fluorescence images are shown. Scale bar, 2 μm. Magnification: 100×. (B) Cells from three separate experiments (300 total) were examined to calculate the percentage of GFP‐Atg8 puncta that colocalize with Ape1‐RFP puncta. Error bars represent SEM; *n* = 3; unpaired Student's *t* test. (C, D) The translocation of Atg1‐GFP and Ape1‐RFP were examined in WT and *pbs2Δ* cells 1 h after nitrogen starvation. (C) Fluorescence images are shown. Scale bar, 2 μm. Magnification: 100×. (D) Cells from three separate experiments (300 total) were examined to calculate the percentage of Atg1‐GFP puncta that colocalize with Ape1‐RFP puncta. Error bars represent SEM; *n* = 3; unpaired Student's *t* test. (E, F) The translocation of Atg17‐GFP and Ape1‐RFP were examined in WT and *pbs2Δ* cells 1 h after nitrogen starvation. (E) Fluorescence images are shown. Scale bar, 2 μm. Magnification: 100×. (F) Cells from three separate experiments (300 total) were examined to calculate the percentage of Atg17‐GFP puncta that colocalize with Ape1‐RFP puncta. Error bars represent SEM; *n* = 3; unpaired Student's *t* test. (G, H) The translocation of Atg18‐GFP and Ape1‐RFP were examined in WT and *pbs2Δ* cells 1 h after nitrogen starvation. (G) Fluorescence images are shown. Scale bar, 2 μm. Magnification: 100×. (H) Cells from three separate experiments (300 total) were examined to calculate the percentage of Atg18‐GFP puncta that colocalize with Ape1‐RFP puncta. Error bars represent SEM; *n* = 3; unpaired Student's *t* test. (I) Atg8 lipidation in WT and *pbs2Δ* cells were tested. Samples were detected by Coomassie staining (CBB) and Western blot with an anti‐Atg8 antibody. (J) The ratio of Atg8‐PE to total Atg8 was calculated. Error bars represent SEM; *n* = 3; *p < 0.05; unpaired Student's *t* test. (K) GFP‐Atg8 proteinase protection in WT and *pbs2Δ* cells were tested. P5 represents the precipitate after centrifugation at 5000 *g*; P10 is the precipitate after centrifugation at 10 000 *g*. (L) The ratio of non‐degraded GFP‐Atg8 was calculated. Error bars represent SEM; *n* = 3; ***p < 0.001; unpaired Student's *t* test.

To investigate the specific stage at which Pbs2 regulates macroautophagy, we did a series of assays. First, GFP‐Atg8 and Ape1‐RFP fluorescence colocalization indicated that PBS2 deletion does not affect the pre‐autophagosomal structure (PAS) localization of Atg8 (Figure 2A,B). Then, the localization of other Atg proteins in PAS were detected. Atg1, Atg13, Atg17 function in the initial step of autophagosome formation; Atg18 forms a complex with Atg2 and functions in autophagosome formation. The results show that colocalization of Atg1, Atg17 or Atg18 with Ape1‐RFP did not differ between WT and *pbs2Δ* cells (Figure 2C–H).

Next, we examined whether Pbs2 affects Atg8 lipidation, which is crucial for the formation and extension of the autophagosome membrane. During earlier stages of macroautophagy, Atg8 is conjugated with phosphatidylethanolamine (PE) to form lipidated Atg8‐PE.^22^ The proportion of lipidated Atg8 (Atg8‐PE) relative to total Atg8 primarily reflects the extent of autophagy blockade specifically at stages following Atg8 lipidation. If autophagy fails to properly proceed through the late stages, lipidated Atg8 generated during the early stages will accumulate significantly. Non‐lipidated Atg8 and lapidated Atg8 (Atg8‐PE) can be separated by urea‐containing SDS‐PAGE.^23^ In WT cells, Atg8‐PE accumulated with prolonged starvation time. In comparison, *pbs2Δ* cells exhibited a more pronounced accumulation of Atg8‐PE (Figure 2I,J). This suggests that Pbs2 does not affect the lipidation of Atg8 or the processes preceding it. Instead, Pbs2 seems to influence the stages following lipidation.

Additionally, we employed the proteinase K protection assay to evaluate the role of Pbs2 in autophagosome closure. During autophagy, autophagosomes form closed vesicles to encapsulate substrates and deliver them to the vacuole. In this assay, only GFP‐Atg8 proteins not protected by fully closed autophagosomes are cleaved into GFP upon proteinase K treatment. When treated with both Triton X‐100 and proteinase K, even mature autophagosomes are degraded, allowing us to assess the proportion of mature autophagosomes. Our findings indicated that 31% of GFP‐Atg8 was protected in WT cells, while this proportion increased to 43% in *pbs2Δ* cells (Figure 2K,L), demonstrating an accumulation of mature autophagosomes. This result, along with data from the Atg8 lipidation assay, suggests the presence of a defect in later‐stage autophagy. This defect likely occurs after the stage of autophagosome closure.

## 4 DISCUSSION

This study explored the role of Pbs2 in non‐selective autophagy triggered by nitrogen starvation. The findings indicate that Pbs2 is essential for autophagy during nitrogen starvation and plays a role in the stages following the closure of autophagosomes. These results enhance our understanding of the biological functions of the Pbs2 kinase in cells. Previous studies have shown that proteins in the HOG pathway, including Pbs2 and Hog1,^24^ can indirectly regulate the phosphorylation of the mitophagy‐specific receptor protein Atg32, thereby controlling the initiation of mitophagy.^12^, ^13^ This provides some evidence for the link between the MAPK cascade and selective autophagy. Many factors involved in mitophagy are also involved in the macroautophagy pathway, such as Hrr25 (HO and Radiation Repair), Bub1 (Budding Uninhibited by Benzimidazole), and Snf1 (Sucrose NonFermenting). Therefore, the relationship between the HOG pathway and macroautophagy was explored. Our study demonstrated that in non‐selective, nitrogen starvation‐induced macroautophagy, Pbs2 but not Hog1 was involved.

Through a series of assays targeting different stages of macroautophagy, we systematically ruled out the possibility that Pbs2 contributes to earlier stages of macroautophagy. The accumulation of Atg8‐PE (Figure 2C,D) and closed autophagosomes (Figure 2E,F) suggested that Pbs2 regulates macroautophagy by affecting the processes following the closure of autophagosomes. This finding adds to current knowledge of the role of Pbs2 in macroautophagy. However, the mechanism of how this protein affects the later stages of macroautophagy remains to be explored. Our findings suggest that Pbs2 likely regulates macroautophagy independently of Hog1. It would therefore be valuable to identify the specific factors, other than Hog1, that mediate Pbs2's role in the later stages of macroautophagy. Studies have suggested that Pbs2 physically interacts with Vam6, a subunit of the HOPS endocytic tethering complex.^25^ Given that previous studies have also demonstrated that the HOPS complex mediates physical tethering and subsequent fusion between autophagosomes and vacuoles, we propose that Pbs2 may phosphorylate Vam6, thereby potentially regulating autophagosome‐vacuole fusion through the HOPS complex.^26^ In this manner, Pbs2 could contribute to the fusion of autophagosomes with vacuoles. Furthermore, given that Pbs2 physically interacts with both Vma1 and Vma6—subunits of the V‐ATPase complex—it is plausible that Pbs2 also influences the degradation stage of autophagy, because the V‐ATPase complex is critical for maintaining the acidic vacuolar environment necessary to activate hydrolases responsible for degrading autophagosomal contents.^27^, ^28^ Based on this, we speculate that Pbs2 may modulate the degradation process of autophagy through its interaction with the V‐ATPase complex. Taken together, these observations suggest that Pbs2 may participate in both the fusion of autophagosomes with vacuoles and the subsequent degradation steps, potentially through its interactions with the HOPS and V‐ATPase complexes, respectively.

## AUTHOR CONTRIBUTIONS

**Jianing Song:** Data curation; formal analysis; investigation; methodology; visualization; writing – original draft; writing – review and editing. **Haolin Zhang:** Formal analysis; investigation; supervision; validation; writing – original draft; writing – review and editing. **Xingyu Cao:** Investigation; visualization. **Zizhang Ren:** Investigation; visualization. **Chao Tian:** Investigation; visualization. **Miao Jia:** Investigation; visualization. **Meiling Wu:** Investigation; visualization. **Xiaoli Wang:** Methodology; resources. **Juan Wang:** Conceptualization; funding acquisition; methodology; project administration; resources; supervision; validation; writing – original draft; writing – review and editing.

## CONFLICT OF INTEREST STATEMENT

Haolin Zhang is a member of AMEM's editorial board and a coauthor of this short communication. To reduce bias, he was excluded from making editorial decisions.

## FUNDING INFORMATION

The National Natural Science Foundation of China (Nos. 32370805 and 31970044).

## ETHICS STATEMENT

This research was conducted in accordance with the ethical standards of Beijing University of Technology.

## Supporting information


Figure S1.
