# Supplementary material for: Pbs2 regulates late‐stage macroautophagy in Saccharomyces cerevisiae
Source: Animal Model Exp Med. 2025 Jun 16;8(7):1321–7. doi: 10.1002/ame2.70042 (PMC12431567; doi:10.1002/ame2.70042)
Supplement: Supplementary file 1 — Figure S1. [file AME2-8-1321-s001.pdf]

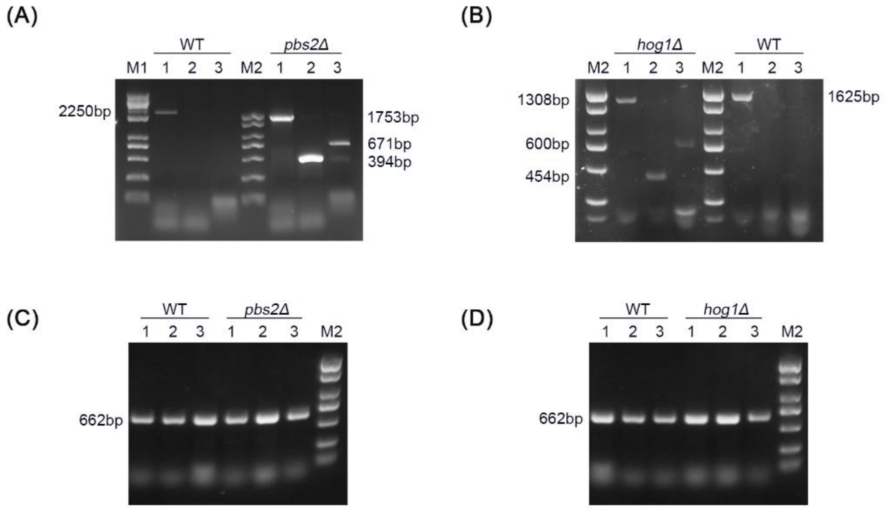

Figure S1. Construction and PCR validation of yeast strains.

(A) PCR validation was conducted to analyze the wild-type (WT) and *pbs2Δ* (PBS2 deletion) strains. The PCR products were resolved on a 1.0% agarose gel. Lane 1 shows the PCR products amplified using primers targeting the flanking sequences of the PBS2 coding sequence. Lane 2 displays the PCR products generated by employing a forward primer specific to the upstream flanking sequence of the PBS2 coding sequence and a reverse primer complementary to the KANMX6 knock-in sequence. Lane 3 presents the PCR products obtained with a forward primer targeting the KANMX6 knock-in sequence and a reverse primer recognizing the downstream flanking sequence of the PBS2 coding sequence. Lane M1 contains a set of 12 DNA marker bands with the following sizes: 10,000 bp, 8,000 bp, 6,000 bp, 5,000 bp, 4,000 bp, 3,000 bp, 2,000 bp, 1,000 bp, 750 bp, 500 bp, 250 bp, and 100 bp. Lane M2 comprises 7 DNA marker bands of the following sizes: 2,000 bp, 1,500 bp, 1,000 bp, 750 bp, 500 bp, 250 bp, and 100 bp.

(B) PCR validation was conducted to analyze the wild-type (WT) and *hog1Δ* (HOG1 deletion) strains. The PCR products were resolved on a 1.0% agarose gel. Lane 1 shows the PCR products amplified using primers targeting the flanking sequences of the HOG1 coding sequence. Lane 2 displays the PCR products generated by employing a forward primer specific to the upstream flanking sequence of the HOG1 coding sequence and a reverse primer complementary to the KANMX6 knock-in sequence. Lane 3 presents the PCR products obtained with a forward primer targeting the KANMX6 knock-in sequence and a reverse primer recognizing the downstream flanking sequence of the HOG1 coding sequence. M2 lanes comprise 7 DNA marker bands of the following sizes: 2,000 bp, 1,500 bp, 1,000 bp, 750 bp, 500 bp, 250 bp, and 100 bp.

(C) PCR was carried out to validate the deletion of the 60 N - terminal amino acids from Pho8 in the context of wildtype (WT) and *pbs2Δ* (PBS2 deletion) strains. Lanes 1, 2, and 3 contained PCR products obtained from 3 independent monoclonal strains, using primers targeting the knock-in sequences. The M2 lane comprises 7 DNA marker bands of the following sizes: 2,000 bp, 1,500 bp, 1,000 bp, 750 bp, 500 bp, 250 bp, and 100 bp.

(D) PCR was carried out to validate the deletion of the 60 N - terminal amino acids from Pho8 in the context of wildtype (WT) and *hog1Δ* (HOG1 deletion) strains. Lanes 1, 2, and 3 contained PCR products obtained from 3 independent monoclonal strains, using primers targeting the knock-in sequences. The M2 lane comprises 7 DNA marker bands of the following sizes: 2,000 bp, 1,500 bp, 1,000 bp, 750 bp, 500 bp,

250 bp, and 100 bp.
